# Application of the Bidirectional Encoder Representations from Transformers Model for Predicting the Abbreviated Injury Scale in Patients with Trauma: Algorithm Development and Validation Study

**DOI:** 10.2196/67311

**Published:** 2025-05-29

**Authors:** Jun Tang, Yang Li, Keyu Luo, Jiangyuan Lai, Xiang Yin, Dongdong Wu

**Affiliations:** 1 Department of Information, Daping Hospital, Army Medical University, No.10 Daping Changjiang Branch Road, Yuzhong District, Chongqing, 400042, China, 86 18302302369; 2 Department of Emergency Medicine, Medical Center of Trauma and War Injury, Daping Hospital, Army Medical University, Chongqing, 400042, China; 3 National Key Laboratory of Trauma and Chemical Poisoning, Chongqing, 400042, China; 4 Department of Orthopedics, Daping Hospital, Army Medical University, No.10 Daping Changjiang Branch Road, Yuzhong District, Chongqing City, Chongqing, 400042, China; 5 Department of Traumatic Surgery, School of Basic Medicine, Army Medical University, Chongqing, 400042, China

**Keywords:** trauma, abbreviated injury scale, deep learning, diagnostic information, transformer model, validation study

## Abstract

**Background:**

Deaths related to physical trauma impose a heavy burden on society, and the Abbreviated Injury Scale (AIS) is an important tool for injury research. AIS covers injuries to various parts of the human body and scores them based on the severity of the injury. In practical applications, the complex AIS coding rules require experts to encode by consulting patient medical records, which inevitably increases the difficulty, time, and cost of evaluation of patient and also puts higher demands on the workload of information collection and processing. In some cases, the sheer number of patients or the inability to access detailed medical records necessary for coding further complicates independent AIS codes.

**Objective:**

This study aims to use advanced deep learning techniques to predict AIS codes based on easily accessible diagnostic information of patients to improve the accuracy of trauma assessment.

**Methods:**

We used a dataset of patients with trauma (n=26,810) collected by the Chongqing Daping Hospital between October 2013 and June 2024. We mainly selected the patient’s diagnostic information, injury description, cause of injury, injury region, injury types, and present illness history as the key feature inputs. We used a robust optimization Bidirectional Encoder Representations from Transformers (BERT) pretraining method to embed these features and constructed a prediction model based on BERT. This model aims to predict AIS codes and comprehensively evaluate its performance through a 5-fold cross-validation. We compared the BERT model with previous research results and current mainstream machine learning methods to verify its advantages in prediction tasks. In addition, we also conducted external validation of the model using 244 external data points from the Chongqing Emergency Center.

**Results:**

The BERT model proposed in this paper performs significantly better than the comparison model on independent test datasets with an accuracy of 0.8971, which surpassed the previous study by 10 % points. In addition, the area under the curve (AUC value of the BERT model is 0.9970, and the *F*_1_-score is 0.8434. In the external dataset, the accuracy, AUC, and *F*_1_-score results of the model are 0.7131, 0.8586, and 0.6801, respectively. These results indicate that our model has high generalization ability and prediction accuracy.

**Conclusions:**

The BERT model we proposed is mainly based on diagnostic information to predict AIS codes, and its prediction accuracy is superior to previous investigations and current mainstream machine learning methods. It has a high generalization ability in external datasets.

## Introduction

### Background

With the frequent occurrence of traffic crashes and the intensification of natural disasters, injuries have become the main cause of morbidity and mortality worldwide. According to the World Health Organization’s (WHO) 2022 report [1], approximately 4.4 million people die, and tens of millions endure from nonfatal injuries every year due to such incidents.

The Abbreviated Injury Scale (AIS) [2] is the most widely used injury severity coding system, developed and periodically refined by the AIS Committee under the Association for the Advancement of Automotive Medicine (AAAM). AIS serves as the foundation for several severity scoring systems, such as the Injury Severity Score (ISS) [3], the Maximum Abbreviated Injury Score [4], and the New Injury Severity Score [5]. Since 2008, the AIS score or ISS score has been used as a criterion for evaluating trauma centers in various countries and has now developed into a globally recognized trauma scoring system.

However, the AIS coding system is a highly refined and complex scoring system that covers injuries to various parts of the human body and scores them based on the severity of the injury. In practical applications, AIS codes often rely on the subjective judgment and rich clinical experience of medical professionals, which may lead to certain coding differences between different medical institutions or personnel. While advances in trauma care have improved overall outcomes, significant disparities persist across sociodemographic groups. Low-income populations experience 38% longer prehospital delays for penetrating injuries compared with high-income counterparts [6], potentially biasing AIS severity assessments due to delayed clinical documentation. This dual challenge of subjective variability and systemic bias further increases the difficulty of accurate AIS code prediction.

The application of artificial intelligence (AI) models in medicine is increasing and many are based on AIS codes to predict mortality and prognosis outcomes [7,8]. While few studies have used diagnostic-related information to predict AIS codes. Although the neural machine translation (NMT) [9] model uses International Classification of Diseases (ICD) codes and other relevant information to predict AIS codes, the accurate acquisition of ICD codes itself necessitates substantial coding effort and also depends on detailed medical records and other clinical information during the patient’s diagnostic process. Therefore, this also puts higher demands on the workload of information collection and processing. To overcome these shortcomings, we hope to use advanced deep learning (DL) techniques to directly predict AIS codes based on easily accessible diagnostic information, thereby improving the accuracy of trauma assessment for patients.

Therefore, we aim to use patient with trauma data from Chongqing Daping Hospital from October 2013 to June 2024 to construct a Bidirectional Encoder Representations from Transformers (BERT) [10] model based on DL for predicting the AIS codes corresponding to specific trauma. In this model, we use the patient’s diagnostic information as the main input feature and compare it with the NMT model from previous research.

### Related Work

In recent years, AI technology has been frequently used to discover complex correlations between various features in medical applications [11-13], such as individual injuries and mortality [14]. Lee et al [15] developed an ensemble model based on deep neural networks, incorporating the ICD, triage scale, procedure codes, and other clinical features as inputs to predict in-hospital mortality among patients with physical trauma. This model achieved an area under the curve (AUC) of up to 0.9507, outperforming advanced predictive models such as AdaBoost and XGBoost. Kang et al [8] created an AI algorithm grounded in DL models, leveraging the AIS codes to predict in-hospital mortality. By comparing their model with conventional ISS and New Injury Severity Score systems, they demonstrated the superior accuracy and AUC value of their proposed model. Tran et al [16] used ICD-10 codes and machine learning (ML) algorithms to develop a mortality prediction model via the National Trauma Data Bank. A comparison of its performance with that of logistic regression, ISS, and Trauma Mortality Prediction Model (TMPM-ICD10) validated that their XGBoost–based ML model exhibited superior performance. In terms of AIS code prediction, Hartka et al [9] proposed the use of an NMT model to convert ICD codes into AIS codes and compared its accuracy in assessing injury severity with that of two established conversion methods: the ICD-AIS map [17] and the ICD Programs for Injury Classification in R (ICDPIC-R) package [18]. Their results demonstrated that the NMT model achieved the highest accuracy across all injury severity classifications.

In the past few years, advanced pretrained language representation models such as BERT, Robustly Optimized BERT Pretraining Approach (RoBERTa), and HFL (a Chinese BERT pretraining model) have made remarkable breakthroughs in the field of natural language processing, demonstrating significant performance gains in various tasks such as text classification, sentiment analysis, and question answering [19]. However, to our current knowledge, despite the increasingly widespread application of DL techniques in the field of medical information processing, DL methods with pretrained language representation models have not yet been widely used for predicting AIS codes. Although the NMT model has shown some accuracy in predicting AIS codes, the AIS coding system, as a standardized tool for assessing the severity of injuries, occupies an important position in trauma medicine and emergency medicine. It can provide objective and comparable injury assessment for clinical doctors based on the specific injury situation of patients, which is of great significance for guiding treatment decisions and evaluating prognosis.

## Methods

### Patients and Dataset

The Chongqing Daping Hospital Trauma Database contains data about patients’ diagnostic information, injury description, age, sex, place of injury, cause of injury, external cause code 1 (ECode1), external cause code 2 (ECode2), injury region, injury types, present illness history, and AIS codes, where the AIS codes are based on the AIS2015 version [20], provided by professionally trained doctors according to the specific injury situation of the patient. To ensure the accuracy of the coding, the hospital has adopted a dual coding system: one doctor is responsible for preliminary coding, while the other doctor conducts follow-up checks.

The Daping Hospital Trauma Database contains data from 26,810 patients registered between October 1, 2013 and June 30, 2024 with the exclusion criteria of (1) patients transferred to another hospital, (2) patients who died in the emergency department before admission to the ICU or general ward, (3) data in the Daping Hospital Trauma Database with a feature loss rate ≥30%, (4) samples with any missing AIS codes or diagnostic information, and (5) data with less than 30 AIS code categories. According to the exclusion criteria, 13,216 pieces of data met the requirements. In addition, we also used an external dataset of 244 Chongqing Emergency Centers that met the inclusion and exclusion criteria.

We divided the Daping Hospital Trauma Database dataset into training data and testing data. The training dataset includes data from October 1, 2013 to December 31, 2022, which is used to train our model to learn the mapping relationship from input features to target variables. The test dataset includes data from January 1, 2023 to June 30, 2024. This partitioning dataset method has multiple benefits: the test dataset uses the most recent data, which can better evaluate the model’s adaptability. By having the model learn historical data during the training phase and then face different but relevant data during the testing phase, it can encourage the model to learn more generalized features, which helps improve the accuracy of predicting future unknown data.

The number of training, testing, and external datasets is 10,827, 2389, and 244, respectively, with 337 types of AIS codes included in the training dataset, 332 types in the testing dataset, and 83 in the external dataset. In the training dataset, the number of AIS code 853161.3 is the highest, reaching 475 (accounting for 4.38%), while the number of AIS code 854221.2 is the lowest, at 25 (accounting for 0.23%). In the test dataset, the number of AIS code 853161.3 is also the highest, at 119 (accounting for 4.98%), while the number of AIS code 910200.1 is the lowest, at 6 (accounting for 0.25%). In the external dataset, the number of AIS code 853161.3 is also the highest, reaching 10 (accounting for 4.10%), while the number of AIS code 856151.2 is the lowest, at 1 (accounting for 0.41%). The injuries covered by our dataset are mainly concentrated in areas such as the skin, limbs, and head and neck, especially those types of injuries that are most common in practical work and have a direct impact on clinical decision-making and treatment plans.

The testing dataset (n=2389) was used only to independently test our developed model and not for training or internal validation. We first performed a 5-fold cross-validation using the training data to prevent overfitting. The training dataset (n=10,827) was randomly shuffled and stratified into 5 equal groups with 4 groups used for training and 1 group used for validation. This process was repeated 5 times by shifting the internal validation group. Then, the overall performance of the model was evaluated through independent testing data. Finally, the generalization ability of the model was validated through multicenter validation using external data.

### BERT Prediction Model Development

#### BERT Model Architecture

**Figure 1.**
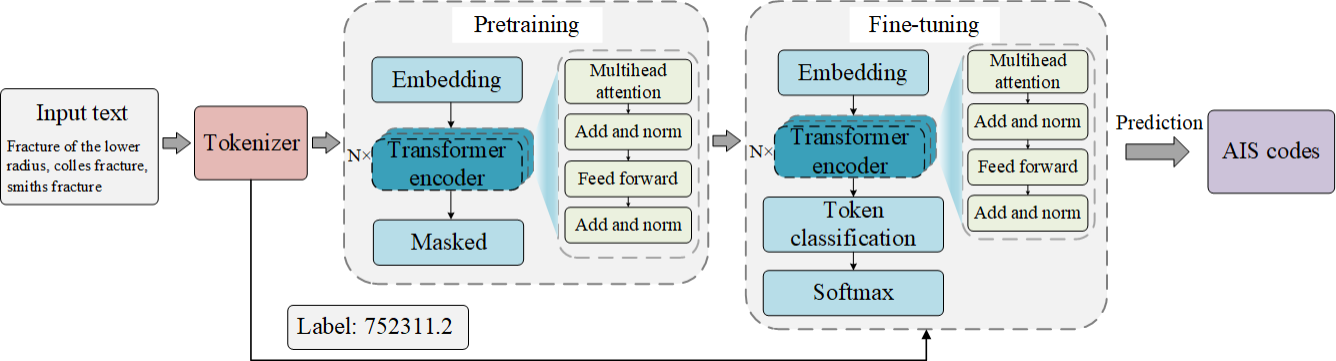
The Bidirectional Encoder Representations from Transformers model architecture. AIS: Abbreviated Injury Scale.

As shown in Figure 1, we developed a DL–based BERT model for predicting AIS codes. The model uses the masked language modeling technique of the pretrained model RoBERTa [10] to learn contextual information about data of patient with trauma. This approach can capture both forward and backward contextual information of the input sequences to achieve a deeper understanding of input textual data.

To provide further clarity on the data used in our model, we have included a Multimedia Appendix 1 with examples of model inputs and outputs. These examples illustrate the structure of the data and how our model processes it to generate AIS scores.

##### BERT Pretraining

Our BERT model is a pretraining model based on a modified version of the RoBERTa model, and we similarly carry out in-depth optimizations of the BERT model, including the implementation of dynamically tuned masking strategies and enhanced text encoding processing. The significant advantage of these optimizations is that by randomly performing masking operations on the input data, different masking patterns are used for the same training data in different training epochs, thus effectively increasing data diversity during model training without the need to expand the training dataset. In addition, by adopting a larger batch size, more training data, and longer training time, the BERT model can learn richer linguistic features and potential patterns in the data, which significantly improves the prediction performance of the model.

Specifically, the vocabulary list used for training includes the patient’s diagnostic information, injury description, age, sex, place of injury, cause of injury, procedure codes (ECode1 and ECode2), injury region, injury types, and present illness history, as well as the necessary special tokens (eg,<CLS_TOKEN>,<SEP_TOKEN>,<PAD_TOKEN>, and <MASK_TOKEN>). The entire text sequence is treated as a sentence with the sequence being identified by the start token <CLS_TOKEN> and the end token <SEP_TOKEN>;<PAD_TOKEN> is used to pad the sequence to a uniform length, and <MASK_TOKEN> is used for masking. We chose the masked language modeling method for training to accommodate the need for contextual understanding in AIS code prediction. During training, we randomly perturbed 15% of the trauma diagnostic information elements in the input sequences, similar to the RoBERTa setup, where 80% of the tokens of these 15% selected elements were replaced with <MASK_TOKEN>, requiring the model to predict the correct AIS codes at these <MASK_TOKEN> locations during training, 10% of the tokens were replaced with other trauma diagnostic information randomly selected to increase the difficulty of training and generalization, and the remaining 10% remained unchanged and served as positive samples for model learning. During pretraining, the model predicts what kind of residue it is in the masked position. For each batch, the loss is defined as:


$$Loss=\frac{-1}{|batch|}\sum_{seq\in batch}\sum_{i\in mask}log\;\hat{p}\left(s_{i}|S\right)$$

where $\hat{p}(s_{i}|S_{\backslash mask})$ represents the probability that the model predicts the element $s_{i}$ at the ith masked position, given all the sequence information except for the masked positions.

##### BERT Fine-Tuning

We consider the task of AIS code prediction as a multivariate labeling classification task, where the BERT model is used to predict the AIS codes for each instance in the input sequence. To accomplish this, we map AIS code categories to unique integer labels, which are used as supervised learning objectives. We add a multivariate classification header on top of the pretrained model, whose output dimension matches the number of AIS codes categories. During training, the BERT model receives input sequences and extracts contextually relevant feature representations. These features are then passed to the multivariate classification header to generate predicted probabilities corresponding to each AIS code category. We use a cross-entropy loss function to compute the difference between the predicted probability distribution and the true AIS code label. The loss during fine-tuning is defined as:


$$L=\frac{-1}{N}\sum_{i=1}^{N}log\left({\hat{y}}_{i,y_{i}}\right)$$

where $y_{i}$ is the true category index of the ith sample, and ${\hat{y}}_{i,y_{i}}$ is the probability that the ith sample predicted by the model belongs to category $y_{i}$.

### BERT Model Configuration

After a careful hyperparameter search, we determined the optimal model configuration: an 8-layer BERT architecture including an input layer, six 384-unit hidden layers, and an output layer, which together form the encoder-decoder transformer components with 5 transformer blocks. In the process of determining the 8-layer architecture, we conducted subsequent experiments and tried different configurations of BERT models with 4, 8, and 12 layers and comprehensively evaluated their performance. The results showed that the 8-layer architecture demonstrated excellent performance on multiple evaluation metrics, so we ultimately chose this architecture for our research. The model is also configured with 12 attention heads and an embedding size of 128. During optimization, we used the Adam optimizer to adjust the model weights with a learning rate of 0.0001, a batch size of 64, and the Gaussian Error Linear Unit as the activation function. To further reduce the number of parameters and computational costs, we implemented low-rank factorization techniques in the embedding layer.

The BERT model was trained via early stopping with training concluding after the validation loss did not improve after 10 epochs. The model was trained on a computing cluster with 48 GB of memory and one Graphics Processing Unit (NVIDIA RTX A6000). We implemented the model through the Pytorch framework.

### Evaluation Methods

We use accuracy, AUC, and *F*_1_-scores as evaluation metrics. Because our prediction task is a multiclass problem and all categories of AIS codes are considered equally important, we chose the macro averaging method when calculating AUC and *F*_1_-scores to ensure that the model’s performance across all AIS code categories is comprehensively reflected.

### Comparison Method

To validate the performance of our BERT model, we selected a series of representative methods for comparison. Specifically, we used previous research (NMT) and current mainstream ML methods, including K-nearest neighbors (KNN), multilayer perceptron (MLP), XGBoost, AdaBoost, and decision tree (DT). The following is a detailed introduction to these methods:

#### Neural Machine Translation

The NMT model proposed by Hartka et al [9] is a DL technique commonly used for human language translation. The model is implemented using OpenNMT, an open-source toolkit developed by the Harvard NLP team and SYSTRAN for NMT, to convert ICD codes into exact AIS codes. This paper shares the same goals and tasks as their work. However, accurately obtaining ICD codes not only requires a lot of coding work but also relies on detailed medical records and other clinical information during the patient’s diagnostic process. In contrast, the BERT model mainly relies on easily accessible diagnostic information to predict specific AIS codes. The NMT model and the BERT model both adopt a similar Transformer architecture. In their experimental configuration, the NMT model includes 6 hidden layers with 512 units, 8 attention heads, a loss rate of 0.1, weights adjusted by the Adam optimizer, learning rate decay determined by Noam decay, and classification cross entropy used as the training loss function.

#### Machine Learning

For ML methods, we use Word2Vec word embedding technology to convert text data into a format suitable for ML algorithm processing.

##### K-Nearest Neighbor

KNN is a simple but effective classification algorithm. It is based on distance metrics such as Euclidean distance to find the k samples in the training set that are most similar to the test samples and predicts the category of the test samples based on the categories of these neighbors. In our experiment, the value of k was set to 3.

##### Multilayer Perceptron

MLP is a feedforward neural network consisting of an input layer, a hidden layer, and an output layer. It approximates complex functional relationships through multilayer nonlinear transformations. In our experiment, MLP used 2 hidden layers with the first layer having 20 neurons and the second layer having 50 neurons.

##### XGBoost

XGBoost is an ensemble learning method based on gradient boosting, which constructs strong classifiers by combining multiple weak classifiers. XGBoost has achieved significant performance improvements in multiple fields, especially when dealing with large-scale datasets and high-dimensional features. In our experiment, we used multiclass log loss as an evaluation metric, which is a commonly used choice in multiclass classification problems.

##### AdaBoost

AdaBoost is an adaptive boosting algorithm that constructs strong classifiers by adjusting the weights of each weak classifier. AdaBoost has demonstrated strong performance in handling classification tasks, especially when dealing with imbalanced datasets. In our experiment, a DT stump (with a depth of 1) was used as the weak classifier.

##### Decision Tree

DT is an intuitive classification and regression method. It generates decision paths through a series of conditional judgments, thereby achieving classification or regression prediction of samples. In our experiment, the default Gini impurity was used as a measure of splitting quality, and the model was constructed by recursively segmenting the feature space.

### Ethical Considerations

This study is an observational study; the data used were reviewed and approved by the Internal Review Board of Chongqing Daping Hospital (approval number: 2024_219), and informed consent from patients was exempted.

## Results

### BERT Model Training

The Daping Hospital dataset used in this study contains a total of 13,216 records, including 10,827 in the training dataset and 2389 in the independent testing dataset. In addition, we obtained 244 external data from the Chongqing Emergency Center. Table 1 provides a detailed list of the demographic characteristics and injury status of these datasets. In both the training and testing datasets, the IQR of age is 33 years. In terms of gender distribution, males accounted for 62.5%, 59.3%, and 63.9% of the training, testing, and external datasets, respectively. In terms of injury causes, the most common were falls (accounting for 57.2%, 57.9%, and 41.0% in the 3 datasets) and traffic crashes (accounting for 14.4%, 16.6%, and 43.9%, respectively). To reflect the distribution of severity data for single injuries, we presented the severity distribution of individual injuries based on the data after the decimal point of the AIS code. Our dataset mainly includes data for mild, moderate, and severe injuries. It is worth noting that in all 3 datasets, moderate injuries account for the highest proportion, at 45%, 51.5%, and 49.6%, respectively.

**Table 1.** Demographic and injury characteristics of patients with trauma in the datasets (N=13460).

Variables	Training dataset (2013/10‐2022/12)	Testing dataset (2023/01‐2024/06)	External dataset (2023/09‐2023/10)
Total number of patients	10,827	2389	244
Age range (years), IQR	1‐102 (33)	3‐98 (33)	14‐97 (31)
Males (%)	6771 (62.5)	1419 (59.3)	156 (63.9)
**Mechanism of injury, n (%)**			
Traffic crash	1557 (14.4)	397 (16.6)	107 (43.9)
Falls	6189 (57.2)	1382 (57.9)	100 (41.0)
Blunt	123 (1.1)	80 (3.3)	22 (9.0)
Sports injury	329 (3.0)	140 (5.9)	10 (4.1)
Other	2629 (24.3)	390 (16.3)	5 (2.0)
**Severity of injury, n (%)**			
Mild injury	2468 (22.8)	392 (16.4)	40 (16.4)
Moderate injury	4870 (45.0)	1231 (51.5)	121 (49.6)
Severe injury	3489 (32.2)	766 (32.1)	83 (34.0)

### Overall Predictive Performance of the BERT Model

We first compared the performance of the BERT model with the NMT model and several advanced ML models, including KNN, MLP, XGBoost, AdaBoost, and DT. Comparison results on our independent test dataset are shown in Table 2. We used accuracy, AUC, and *F*_1_-scores as evaluation metrics. It is worth noting that since the NMT model only provides prediction results for AIS code accuracy, we only present the accuracy of NMT in Table 2. For other metrics not provided by the NMT model, we uniformly use the symbol “—” for annotation.

The performance of our proposed BERT model is significantly better than all comparison models across all indicators. Specifically, the accuracy of the BERT model is as high as 0.8971, while the accuracy of the NMT model is only 0.7380 with a difference of over 10 % points between the two. In addition, the AUC value of the BERT model is 0.9970, and the *F*_1_-score is 0.8434. Among all the ML methods compared, the DT method achieved excellent performance in accuracy and AUC of 0.8506 and 0.9945, respectively, and the XGBoost method achieved the best results in the *F*_1_ index at 0.7586, but they still failed to surpass the performance of our BERT model. These comparative experiments fully demonstrate that our BERT model has high prediction accuracy.

**Figure 2.**
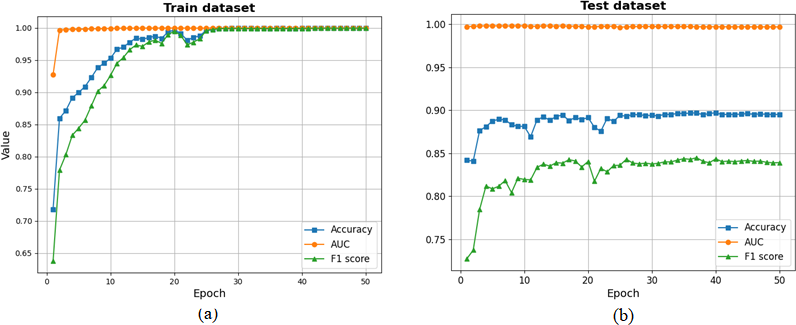
The visualization curve of Bidirectional Encoder Representations from Transformers model’s predictive performance on training and testing datasets. AUC: area under the receiver operating characteristic curve.

Figure 2 shows the training curve of the BERT model with the (**A**) and (**B**) graphs displaying their results on the training and testing datasets, respectively. The x-axis represents epochs and is set to 50, whereas the y-axis represents the values of accuracy, AUC, and *F*_1_-score. The figure shows that the BERT model tends to be stable in all the metrics when the training or test dataset reaches epoch 26. The test dataset shows that the model performs well in terms of overall prediction accuracy and accurately classifies samples into the correct categories. For both the training and testing datasets, the AUC is close to 1, and high AUC values further demonstrate the model’s strong ability to distinguish between positive and negative samples, maintaining excellent performance at almost all possible classification thresholds.

To demonstrate the performance advantage of our fine-tuned BERT model, we compared it with the base version of BERT (BERT-base) and the pretrained model HFL/chinese-roberts-wwm-ext [21]. The comparison results are shown in Table 3. Compared to the BERT-base and HFL models, the BERT model exhibits higher performance in accuracy, reaching 0.8971, while also significantly leading in the *F*_1_-score, at 0.8434. The results of these two indicators are 2.99% and 7.54% higher than the HFL model, respectively, indicating that our BERT model achieves a better balance between accuracy and recall in classification tasks. Although the BERT is not significantly different from HFL and BERT-base on AUC, its advantages in key evaluation metrics highlight BERT’s superior overall performance in handling specific tasks in this paper.

**Table 2.** Prediction results of the Bidirectional Encoder Representations from Transformers model and comparative model in the test dataset.

Model	Accuracy	AUC^a^	*F*^_1_^-scores
NMT^b^	0.7380	NA^c^	NA
KNN^d^	0.7935	0.9414	0.6879
MLP^e^	0.8064	0.9886	0.6194
XGBoost^f^	0.8374	0.9937	0.7586
AdaBoost^g^	0.8506	0.9860	0.7050
DT^h^	0.8506	0.9945	0.7049
BERT^i^	0.8971	0.9970	0.8434

a AUC: area under the curve.

b NMT: neural machine translation.

c NA: not available.

d KNN: K-Nearest Neighbor.

e MLP: multilayer perceptron.

f XGBoost: Extreme Gradient Boosting.

g AdaBoost: adaptive boosting

h DT: decision tree.

i BERT: Bidirectional Encoder Representations from Transformers

**Table 3.** Comparison between our Bidirectional Encoder Representations from Transformers model and other pretrained models in the test dataset.

Model	Accuracy	AUC^a^	*F*_1_-scores
BERT-base^b^	0.8559	0.9971	0.7284
^c^HFL	0.8672	0.9973	0.7680
BERT	0.8971	0.9970	0.8434

a AUC: area under the curve.

b BERT: Bidirectional Encoder Representations from Transformers.

c HFL: a Chinese BERT pretraining model.

### Ablation Study

To verify the maximum contribution of specific input data feature combinations to the model, we designed a series of ablation studies. The input features of the original experiment include patients’ diagnostic information, age, sex, injury description, place of injury, cause of injury, procedure codes (ECode1 and ECode2), injury region, injury types, and present illness history.

First, we conducted a single-factor ablation study to observe the impact of removing each feature item one by one on the performance of the model. The experimental results show that diagnostic information and injury description are the most significant data types that affect the performance of the model. Based on the findings of the single-factor ablation study, we further designed the following multifactor ablation study to explore the importance of diagnostic information and injury description in feature combination. The specific results are shown in Table 4.

As shown in Table 4, after removing diagnostic information, the accuracy and *F*_1_-score of the model significantly decreased. Although the AUC remained high, it also decreased, indicating that diagnostic information features have a significant impact on the performance of the model. After removing the injury description, the performance of the model also decreased, but the decrease was smaller compared to removing diagnostic information. The ablation study demonstrated that diagnostic information contributed the most to the model, followed by the injury description, and the feature combination we used achieved the best results.

**Table 4.** Results of ablation study in the test dataset.

Model	Accuracy	AUC^a^	*F*_1_-scores
Diagnostic information removed^b^	0.6033	0.9523	0.4668
Injury description removed^c^	0.8888	0.9875	0.8047
Diagnostic information and injury description removed^d^	0.6014	0.9438	0.4699
BERT^e^	0.8971	0.9970	0.8434

a AUC: area under the curve.

b Diagnostic information removed: Remove diagnostic information based on all basic features.

c Injury description removed: Remove the injury description based on all basic features.

d Diagnostic information and injury description removed (simultaneously remove diagnostic information and injury description): Based on all basic features, simultaneously remove diagnostic information and injury description.

e BERT: Bidirectional Encoder Representations from Transformers.

### External Validation

External validation of the constructed BERT model was conducted using data from 244 patients with trauma at Chongqing Emergency Center. This external dataset has a similar data structure to the training dataset, containing a total of 83 AIS code categories. The experimental results of the external dataset are shown in Table 5 with accuracy, AUC, and *F*_1_ of 0.7131, 0.8586, and 0.6801, respectively. Compared with the test dataset, the performance of the BERT model slightly decreases on external datasets, which may be due to differences in data distribution between different medical institutions. But the overall performance is still satisfactory, indicating that the BERT model has strong generalization ability.

**Table 5.** Validation results of Bidirectional Encoder Representations from Transformers model on external datasets.

Dataset	Accuracy	AUC^a^	*F*_1_-scores
Test dataset	0.8971	0.9970	0.8434
External dataset	0.7131	0.8586	0.6801

a AUC: area under the curve.

## Discussion

### Principal Findings

In this study, we successfully constructed a BERT–based DL model using data of patient with trauma from Chongqing Daping Hospital to predict AIS codes, achieving an accuracy of 89.71%. This model, leveraging patient diagnostic information as primary input features, demonstrated superior performance compared to existing advanced AIS prediction models, including the previously studied NMT framework [9]. Additionally, we validated the model’s high generalization ability using data from an external center, thereby fulfilling our objective of enhancing trauma assessment through DL.

Key innovations such as dynamic masking strategies, low-rank embedding decomposition, and bidirectional contextual modeling enabled the model to capture nuanced clinical semantics while maintaining computational efficiency. Notably, the model directly outputs complete AIS codes—a critical advancement over rule-based methods like the ICD-AIS map [17] and the ICDPIC-R package [18] tools, which lack granular code prediction capabilities. These results underscore the potential of transformer-based architectures to enhance trauma assessment workflows, particularly in scenarios requiring rapid, large-scale injury coding.

Our findings align with emerging evidence supporting transformer models in clinical text processing [10], yet extend prior work by addressing the unique challenges of AIS coding. Unlike NMT models that process sequential tokens independently via recurrent mechanisms [9], the BERT model’s bidirectional attention dynamically links contextual elements of injury descriptions. In addition, the pretrained biomedical embeddings provided higher precision in rare injury terminology recognition compared to NMT’s task-specific training.

The model’s external validation performance further reinforces its clinical utility. While annual variations in trauma patterns typically degrade conventional models, our temporal split testing revealed stable predictive accuracy. This robustness suggests the framework could adapt to shifting trauma trends without frequent retraining. Moreover, direct AIS code generation eliminates the multistep mapping required by ICD-based tools [9,17,18], reducing error propagation risks in mass casualty scenarios where rapid triage coding is critical.

These findings suggest that BERT may become a powerful tool for injury research. Although independent coding of AIS injuries by trained medical professionals and comprehensive medical data remains the gold standard in this field, in some cases, such as when the number of patients is large or detailed medical records are difficult to obtain, independent coding becomes impractical. At this point, given input features that satisfy the model, our BERT model can automatically provide prediction results for AIS codes, providing highly accurate AIS code predictions for individual patients.

### Limitations

Our research has several limitations. First, during the data collection and processing phase, a large amount of data was excluded due to the lack of information on injury description and present illness history, which had a significant impact on the integrity and representativeness of the final dataset. Second, while low-rank decomposition improved efficiency, BERT’s inherent sequence length restrictions (≤512 tokens) may truncate complex trauma descriptions. Third, the Chinese-language training data raises questions about cross-lingual applicability, given known variations in medical terminologies across languages [22]. Finally, as with most DL systems, the model’s black-box nature limits clinical interpretability.

Future studies should address these gaps by (1) integrating multimodal data (eg, imaging reports) to compensate for text incompleteness, (2) benchmarking against large language models with superior few-shot learning capacities, and (3) developing hybrid systems that combine BERT’s predictive power with large language model–driven explainability features.

### Conclusions

The BERT model we propose is mainly based on diagnostic information to predict AIS codes, and its prediction accuracy is superior to existing methods. These findings highlight the potential of advanced AI techniques to enhance clinical decision-making processes and improve the efficiency and accuracy of AIS code prediction.

By automating a task that traditionally requires hours of expert review per case, our framework could democratize high-quality trauma registries in resource-limited settings. Crucially, the model does not seek to replace human coders but provides a scalable adjunct for high-volume scenarios—a balance increasingly advocated in AI-augmented health care [23]. As trauma systems worldwide adopt electronic health records, such tools may transform retrospective coding into a prospective clinical decision aid, ultimately bridging the gap between injury documentation and precision trauma care. Future iterations incorporating multi-institutional data and explainability interfaces could further establish BERT-derived models as indispensable tools in computational trauma epidemiology.

## Supplementary material

10.2196/67311 Multimedia Appendix 1 Model input and output examples.

## Abbreviations

AAAM: Association for the Advancement of Automotive Medicine
AI: artificial intelligence
AIS: Abbreviated Injury Scale
AUC: area under the curve
BERT: Bidirectional Encoder Representations from Transformers
DL: deep learning
DT: decision tree
ECode: external cause code
ICD: International Classification of Diseases
ISS: Injury Severity Score
KNN: K-nearest neighbor
ML: machine learning
MLP: multilayer perceptron
NMT: neural machine translation
RoBERTa: Robustly Optimized BERT Pretraining Approach
WHO: World Health Organization
