# Supplementary material for: Application of the Bidirectional Encoder Representations from Transformers Model for Predicting the Abbreviated Injury Scale in Patients with Trauma: Algorithm Development and Validation Study
Source: JMIR Form Res. 2025 May 29;9:e67311. doi: 10.2196/67311 (PMC12140372; doi:10.2196/67311)
Supplement: Multimedia Appendix 1 [file formative-v9-e67311-s001.docx]

# Multimedia Appendix 1: Model Input and Output Examples

The following table provides two examples of input data for the BERT model. Displayed diagnostic information and patient characteristics for model training and prediction.

| Feature | Example 1 | Example 2 |
| --- | --- | --- |
| Diagnostic information | Fracture of the lower radius, colles fracture, smiths fracture | Femoral neck fracture, other unspecified hip fractures |
| Age | 58 | 83 |
| Sex | Female | Male |
| Injury description | Left wrist joint pain with limited mobility caused by a fall for 1 day | Right hip pain with restricted mobility caused by a fall for 4 days |
| Place of injury | Schools, other institutions, and public management areas | Home |
| Cause of injury | Falling | Falling |
| ECode1 | Fall | Fall |
| ECode2 | Injury caused by unspecified falls | Injury caused by unspecified falls |
| Injury region | Skin or limbs | Skin or limbs |
| Injury types | Blunt injury | Blunt injury |
| Present illness history | The patient accidentally slipped and fell while walking at work one day ago, causing pain in the left wrist joint and limited mobility. They were immediately sent to a local hospital for X-ray examination, which showed a distal fracture of the left radial bone. Surgical treatment was recommended. The patient came to our hospital for further treatment and was admitted to our department as a "distal fracture of the left radial bone". The patient is currently in good spirits, with normal physical strength, normal appetite, normal sleep, no significant changes in weight, normal bowel movements, and normal urination. They have been admitted for further examination and treatment | 4+days ago, the patient accidentally fell while walking and immediately felt pain in the right hip accompanied by inability to walk. They sought medical attention at a local hospital and were considered to have a femoral neck fracture (details unknown). It was recommended to transfer the patient to a higher-level hospital for surgical treatment. In order to seek further treatment, the patient was admitted to our emergency department for a femoral neck fracture. The patient is currently in good spirits, with good physical strength, good appetite, good sleep, no significant changes in weight, normal bowel movements, and normal urination. They have been admitted for further examination and treatment |

The following table shows examples of AIS score outputs corresponding to two sets of input data after being processed by the BERT model.

| Feature | Example 1 | Example 2 |
| --- | --- | --- |
| AIS code | 752311.2 | 853161.3 |
| Explanation | "Fracture of the lower radius, colles fracture, smiths fracture" is classified as moderate severity (2) based on AIS scoring criteria. | "Femoral neck fracture, other unspecified hip fractures" is classified as moderate severity (3) based on AIS scoring criteria. |
